# Pathological findings of stereotactic cardiac radiotherapy for the treatment of ventricular tachycardia in patients with Chagas disease: case series

**DOI:** 10.1093/ehjcr/ytaf655

**Published:** 2025-12-18

**Authors:** Rodrigo Melo Kulchetscki, Léa Maria Macruz Ferreira Demarchi, Cristiano Faria Pisani, Bernardo Salvajoli, João Victor Salvajoli, Mauricio Ibrahim Scanavacca

**Affiliations:** Arrhythmia Unit, Heart Institute (InCor), Hospital das Clínicas, Faculty of Medicine, University of São Paulo, Av. Dr. Enéas Carvalho de Aguiar, 44 – Cerqueira César, São Paulo, SP 05403-000, Brazil; Department of Pathology, Heart Institute (InCor), Hospital das Clínicas, Faculty of Medicine, University of São Paulo, Av. Dr. Enéas Carvalho de Aguiar, 44 – Cerqueira César, São Paulo, SP 05403-000, Brazil; Arrhythmia Unit, Heart Institute (InCor), Hospital das Clínicas, Faculty of Medicine, University of São Paulo, Av. Dr. Enéas Carvalho de Aguiar, 44 – Cerqueira César, São Paulo, SP 05403-000, Brazil; Department of Radiotherapy, Cancer Institute (ICESP), Hospital das Clínicas, Faculty of Medicine, University of São Paulo, Av. Dr. Arnaldo, 251 – Cerqueira César, São Paulo, SP 01255-000, Brazil; Department of Radiotherapy, Cancer Institute (ICESP), Hospital das Clínicas, Faculty of Medicine, University of São Paulo, Av. Dr. Arnaldo, 251 – Cerqueira César, São Paulo, SP 01255-000, Brazil; Arrhythmia Unit, Heart Institute (InCor), Hospital das Clínicas, Faculty of Medicine, University of São Paulo, Av. Dr. Enéas Carvalho de Aguiar, 44 – Cerqueira César, São Paulo, SP 05403-000, Brazil

**Keywords:** Myocardial fibrosis, Radiotherapy, Ventricular tachycardia, Chagas disease, Case series

## Abstract

**Background:**

Stereotactic arrhythmia radiotherapy (STAR) is a novel, non-invasive therapeutic option for managing ventricular tachycardia (VT), including in patients with chronic Chagas cardiomyopathy (CCC). However, the histopathological substrate underlying its antiarrhythmic effect remains poorly defined, particularly in the Chagas population. This study aims to characterize the myocardial tissue changes following STAR in two CCC patients, evaluated at different time points after treatment.

**Case summary:**

Two CCC patients with recurrent VT underwent STAR as part of a multidisciplinary treatment protocol. One patient died 50 days post-STAR, and the other underwent heart transplantation 702 days after the procedure. Myocardial tissue was collected from irradiated and non-irradiated regions. Gross pathology, histological staining (haematoxylin–eosin and Masson’s trichrome), and immunohistochemistry for apoptosis markers (p53, Bcl-2, and caspase-3) were performed and analysed by a cardiac pathologist. Macroscopic analysis showed fibrosis in the STAR-targeted areas. Histological evaluation revealed varying degrees of myocyte damage, including cytoplasmic vacuolization and myocytolysis, more pronounced in the early post-STAR case. Extensive fibrosis was present in both cases, but also observed in non-irradiated areas, reflecting underlying CCC pathology. Immunohistochemistry for apoptosis markers was negative in both patients.

**Conclusion:**

Stereotactic arrhythmia radiotherapy areas present myocardial changes consistent with acute cellular injury and fibrosis in CCC patients treated for VT. However, apoptotic activity was not detected within the analysed timeframe. Fibrosis was found in both irradiated and non-irradiated areas, and differentiating STAR-induced fibrosis from baseline Chagas-related remodelling remains challenging.

Learning points

- Stereotactic ablative radiotherapy (SAR) is a treatment option for ventricular tachycardia (VT), including in patients with Chagas disease.
- Both early and late macro- and microscopic findings following SAR in Chagas disease reveal varying degrees of fibrosis and myocyte damage, but no evidence of apoptotic activity. These findings support a multifactorial mechanism of action of SAR in Chagas disease patients

## Introduction

The use of stereotactic arrhythmia radiotherapy (STAR) for the treatment of recurrent ventricular tachycardia (VT) in patients with structural heart disease is a recent development.^1–3^ In patients with chronic Chagas cardiomyopathy (CCC), a first case report was recently described, showing promising results.^4^

The histological effects of radiotherapy on cardiac muscle, which justify its antiarrhythmic effect, are still under investigation. Case series of explanted hearts from patients who underwent STAR have demonstrated varying degrees of cellular injury and apoptotic markers, with fibrosis generally occurring later, around 6 months post-treatment.^5–7^

Cellular apoptosis is a highly regulated process that plays a crucial role in responding to cellular damage, including that induced by STAR. Among the proteins involved in apoptosis regulation, p53, known as the ‘guardian of the genome,’ is a transcription factor that responds to cellular stress by promoting the activation of pro-apoptotic pathways in response to DNA damage. On the other hand, the Bcl-2 protein family plays an anti-apoptotic role by inhibiting the release of pro-apoptotic factors from the mitochondria, such as cytochrome c, thereby preserving mitochondrial integrity. Caspase-3, in turn, is an enzyme responsible for cleaving various structural and functional cellular proteins, ultimately leading to DNA fragmentation and programmed cell death.^8,9^

As part of a larger pilot study investigating the effects of STAR in CCC, this two-case series presents the only explanted cases within the cohort and examines the histopathological effects of STAR at different post-treatment intervals: one patient with CCC who underwent autopsy 50 days after radiotherapy and another who underwent heart transplantation 702 days post-treatment (*Table 1*)

**Table 1:** Timeline of the patients

Patient	Age (years)	Sex	LVEF (%)	Number of prior CA	Amiodarone dose (mg/day)	Reason for including in STAR protocol	Days after STAR	PTV/ITV (cc)	Cause of death or HTx
1	67	Female	35	0	400	Extreme frailty	50	86.89/25.18	CS secondary to ES
2	60	Male	29	1	400	Epicardial adherence (prior surgically corrected megaoesophagus)	702	82.5/27.3	Failure in inotrope weaning

LVEF, left ventricular ejection fraction; CA, catheter ablation; STAR, stereotactic arrhythmia radiotherapy; PTV, planning target volume; ITV, internal target volume; cc, cubic centimetres; HTx, heart transplant; CS, cardiogenic shock; ES, electrical storm.

## Summary figure

**Figure:**
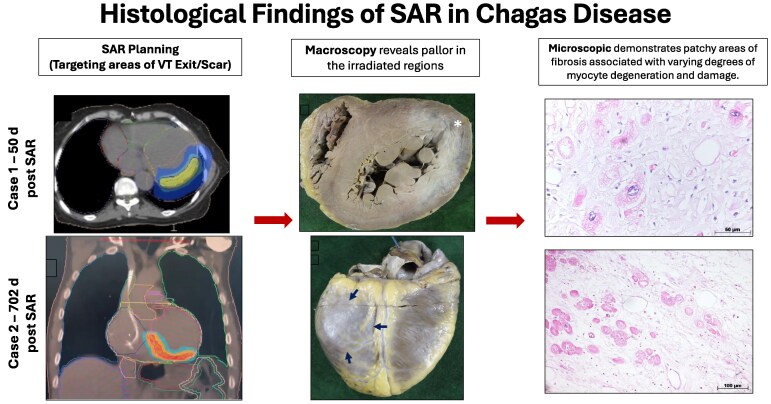


## Methods

### STAR planning protocol

**Figure 1:**
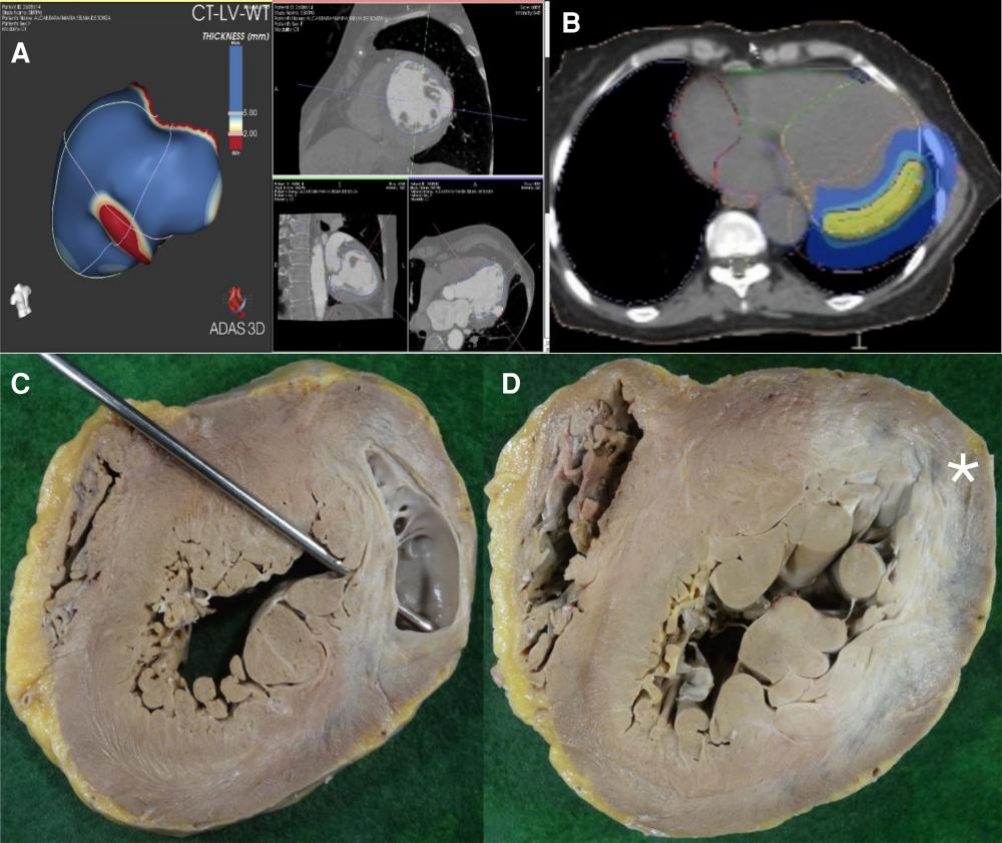
(*A*) 3D reconstruction of coronary computed tomography angiography (ADAS 3D®, Adas3D Medical SL, Barcelona, Spain) showing a finger-like aneurysm in the mid-lateral region of the left ventricle. (*B*) Simulated target area for irradiation in the lateral left ventricle wall, encompassing the aneurysm identified in (*A*). (*C*) Macroscopic view of the left ventricle showing the lateral aneurysm. (*D*) Macroscopic view of the left ventricle highlighting a whitish basal lateral area (*), consistent with the irradiated area, which histologically revealed increased perimysial fibrosis.

**Figure 2:**
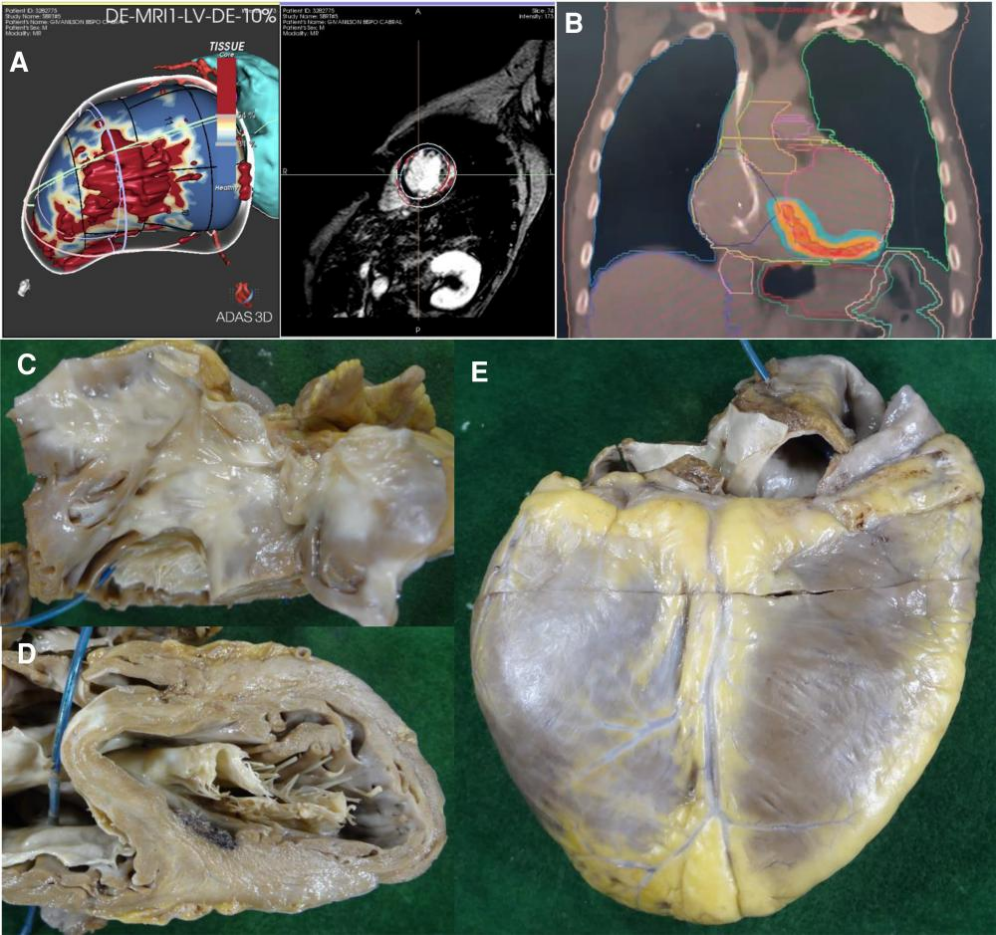
(*A*) 3D reconstruction of coronary computed tomography angiography (ADAS 3D®, Adas3D Medical SL, Barcelona, Spain) showing a scar in the mid-lateral region of the left ventricle. Induced ventricular tachycardias suggested septal and inferior exits; therefore, the irradiated area (*B*) included the entire inferior left ventricle region. (*C*) Macroscopic view of radiofrequency lesions in the right ventricular outflow tract related to previous ablations. (*D*) Area of necrosis in the basal septal left ventricle region—this region underwent both radiofrequency (RF) ablation and stereotactic arrhythmia radiotherapy. (*E*) Whitish area in the mid-basal inferior left ventricle wall, corresponding to the irradiated area (blue arrows). VT, ventricular tachycardia.

The procedure planning was conducted as previously described [4]. In summary: **(1) Recognition of the Arrhythmogenic Area Topography**: an electrophysiological study, which evaluated the morphologies of the induced VT, was performed, along with imaging from coronary computed tomography angiography and/or cardiac magnetic resonance imaging. The processing and 3D reconstruction of the images using the ADAS 3D® software (Adas3D Medical SL, Barcelona, Spain) were instrumental in target delineation. In Case 1, only one induced VT morphology was identified, associated with a finger-like aneurysmal area in the mid-anterolateral wall and apex of the left ventricle (LV) (*Figure 1A*), with the target area identified in this region (*Figure 1B*). In Case 2, a scar was identified extending from the basal septal region to the lateral LV (*Figure 2A*), with VT exits predominantly in the inferoseptal region, leading to the selection of the entire mid-basal inferior LV wall as the target (*Figure 2B*). **(2) Target Identification and Adjacent Organ Risk Assessment:** a 4D computed tomography scan was performed in the radiotherapy department, alongside a study and monitoring of the patient’s respiratory movements. **(3) Multidisciplinary Review:** images from Steps 1 and 2 were analysed by both the radiotherapy and electrophysiology teams to finalize the target definition in the demarcated substrate. **(4) Dosimetry Calculations:** the medical physics team (using Monaco® Elekta | Radiotherapy Treatment Solutions) calculated the energy delivery required for treatment (25 Gy with no dose escalation) while considering the protective limits for adjacent organs such as the lungs and oesophagus **(5) Simulation and Quality Control:** a treatment simulation was performed by the radiation oncologist to ensure quality control before authorizing the actual procedure.

### Morphological evaluation

#### Macroscopic analysis

Hearts obtained through autopsy and heart transplantation were fixed in 10% buffered formalin solution for 24 h and subjected to macroscopic examination.

#### Histological processing, slide preparation, and evaluation

Samples from both irradiated and non-irradiated areas of each heart were collected, routinely processed, and embedded in paraffin blocks. Histological sections of 5 mm were mounted on slides treated with 4% silane and used for histological and immunohistochemical evaluations. Samples were stained using haematoxylin–eosin (HE) and Masson’s trichrome methods. Microscopic analysis was conducted by a cardiac pathologist, and histological changes were described in cardiomyocytes (hypertrophy, myocytolysis, and sarcoplasmic vacuolization) and in the myocardial interstitium (inflammatory infiltrate, fibrosis, and vascular alterations).

#### Immunohistochemical evaluation

Histological sections from both irradiated and non-irradiated areas were subjected to immunohistochemical (IHC) detection using the modified streptavidin–biotin–peroxidase method.^10^ Irradiated areas were defined as those within the planning target volume (PTV). Non-irradiated areas were considered regions receiving a radiation dose of <5 Gy (see Supplementary material online, *Figure S1*). The proteins involved in apoptosis regulation, including p53, Bcl-2, and caspase-3, were analysed using the following primary antibodies: p53 (CONFIRM anti-p53 primary antibody, clone DO-7, Roche Diagnostics, USA; dilution 2.5 μg/5 mL), Bcl-2 (Clone 124—DakoCytomation®, USA; dilution 1:80), and caspase-3 (Polyclonal—DakoCytomation®, USA; dilution 1:400). The IHC detection was identified under optical microscopy by brown staining in the cell nuclei (p53), cell membrane and cytoplasm (Bcl-2), and cytoplasm (caspase-3). The evaluation was performed by a cardiac pathologist.

### Patient 1

A 67-year-old woman with CCC, LV ejection fraction (LVEF) of 35%, significant mitral regurgitation, and a lateral LV aneurysm received a dual-chamber implantable cardioverter-defibrillator (ICD) in 2021 due to syncope, prolonged His-ventricular (HV) interval, and inducible monomorphic VT on electrophysiologic study.

While on amiodarone, she experienced multiple appropriate ICD therapies, mostly with a heart rate of <135 b.p.m. In July 2022, she was admitted with an electrical storm (six shocks in 24 h). After multidisciplinary discussion considering frailty and family preferences, she was enrolled in a STAR research protocol.

Stereotactic arrhythmia radiotherapy was performed in a single session in July 2022 (25 Gy, 80% isodose, 17 min). The PTV was 86.89 cc, and the internal target volume (ITV) was 25.18 cc. She was discharged 5 days later on amiodarone and beta-blockers, asymptomatic. However, 2 weeks later, she returned with recurrent ICD therapies. After an initial conservative approach, she was readmitted within 3 days due to further ICD shocks.

**Figure 3:**
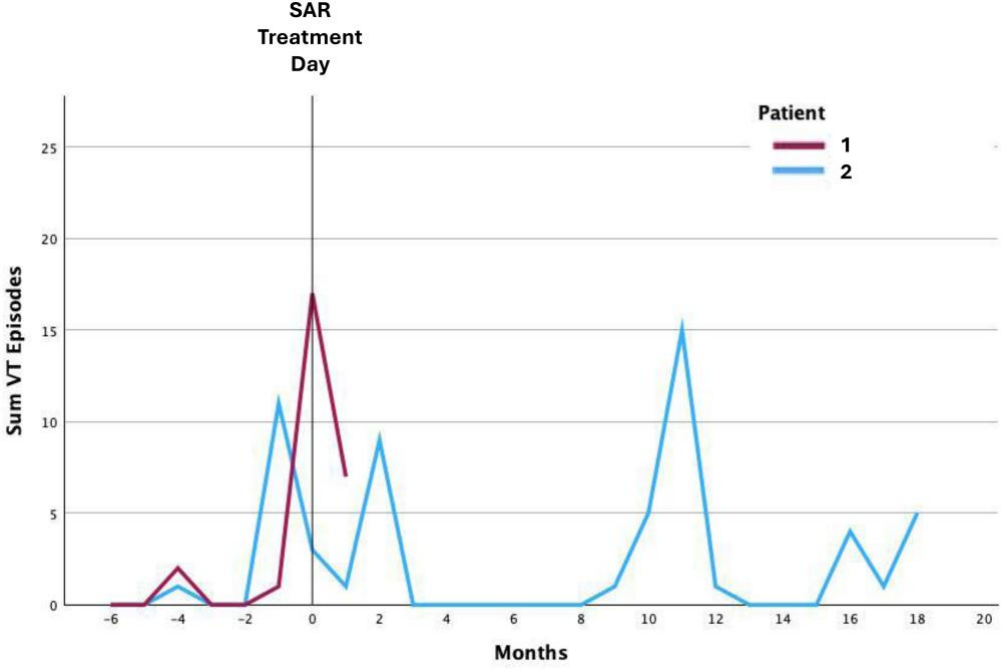
Summary (Sum) of ventricular tachycardia episodes of each patient (Patient 1 with shorter follow-up and Patient 2 in blue) in relation to stereotactic arrhythmia radiotherapy treatment day.

During hospitalization, she had poorly tolerated slow VT (115 b.p.m.), undetected by the ICD, requiring sedation and cardioversion. Her condition worsened to cardiogenic shock with a presumed septic component, requiring intubation and dialysis. She died in September 2022, 50 days post-STAR (*Figure 3*).

Autopsy findings confirmed the presence of aneurysms previously identified in imaging studies, with a significant fibrotic component surrounding the aneurysm in the anterolateral LV wall. This fibrosis was visually identified as a greyish area within the myocardial thickness, corresponding to the irradiated region of the lateral LV wall (*Figure 1C* and *D*).

**Figure 4:**
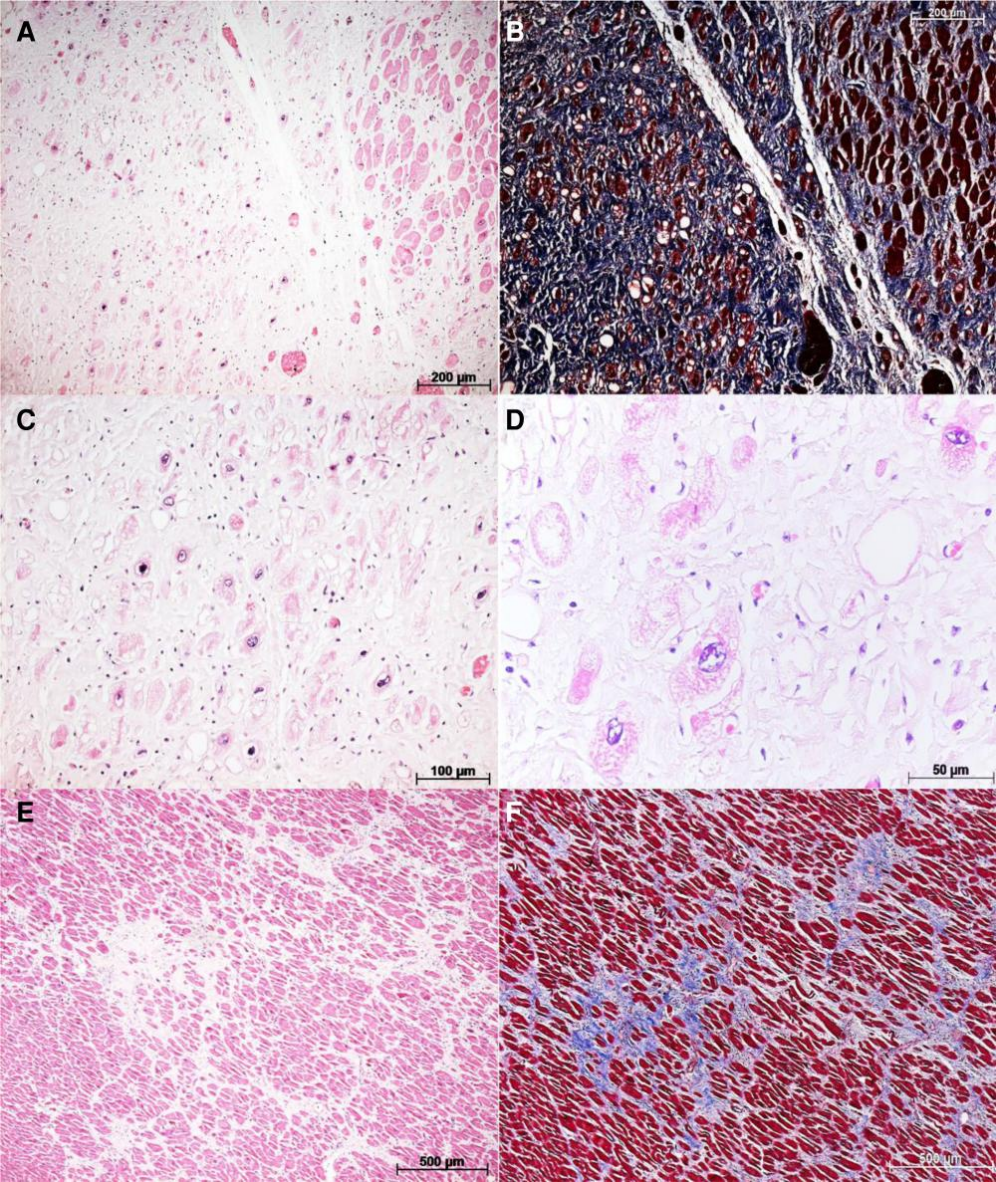
(*A*) Irradiated myocardial area showing cardiomyocyte hypertrophy interspersed with areas of fibrosis. (*B*) Masson’s trichrome staining of (*A*), highlighting the extensive fibrosis (blue) in the lateral wall of the left ventricle. (*C*) Similar to (*A*), but at higher magnification and stained with haematoxylin and eosin, revealing a mild lymphomononuclear infiltrate, associated with areas of myocytolysis and cardiomyocyte vacuolization. (*D*) Similar to (*C*), but at higher magnification. (*E*) Histological section of a non-irradiated area (interventricular septum), suggesting preserved cardiomyocytes with perimysial and endomysial fibrosis to a lesser extent compared to the lateral wall of the left ventricle. (*F*) Similar to (*E*), with fibrosis (blue) better visualized by Masson’s trichrome staining.

**Figure 5:**
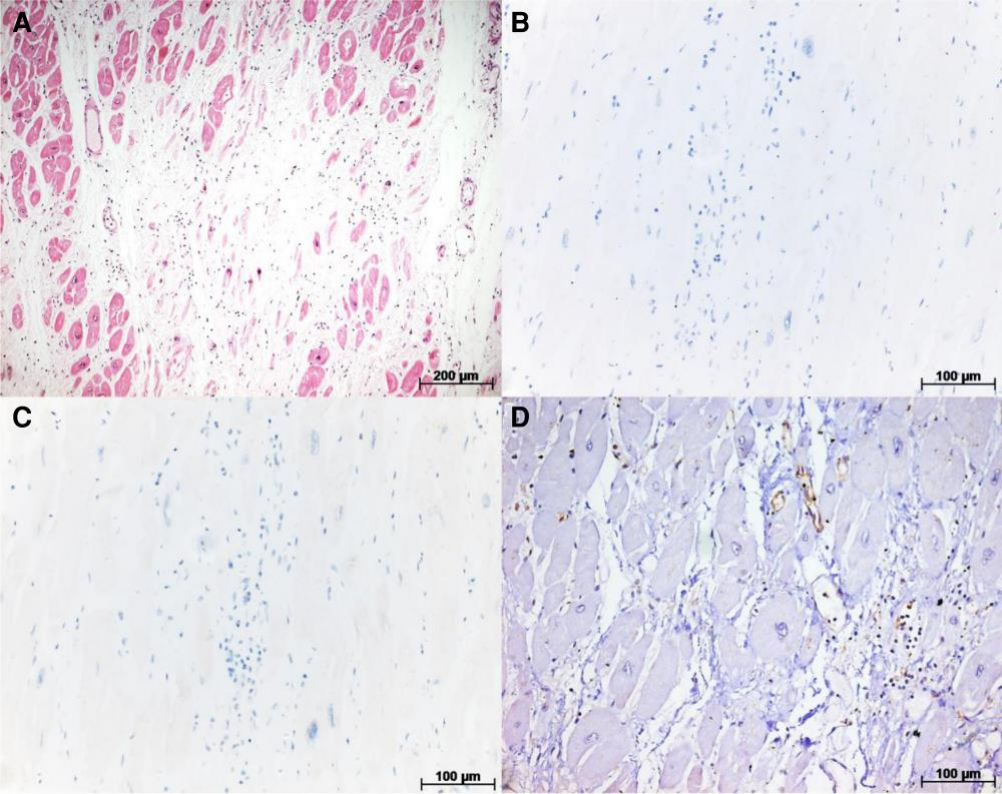
(*A*) Another histological section of the irradiated area stained with haematoxylin–eosin, showing fibrosis associated with mild lymphomononuclear infiltrate, along with some areas of myocytolysis and cardiomyocyte vacuolization. (*B*) Immunohistochemistry for P53—predominantly negative sample. (*C*) Immunohistochemistry for Bcl-2—predominantly negative sample. (*D*) Immunohistochemistry for caspase-3—predominantly negative sample, except for some areas of inflammatory cell uptake, considered a non-specific finding.

Samples were collected from both irradiated and non-irradiated areas (see Supplementary material online, *Figure S2A*). Optical microscopy with HE staining revealed areas of cardiomyocyte vacuolization in the irradiated region, associated with a lymphomononuclear inflammatory infiltrate (*Figures 4A–C* and *5A*). However, inflammatory infiltrates were also observed in non-irradiated areas, but without cardiomyocyte vacuolization, which was consistent with active myocarditis (*Figure 4E*).

Masson’s trichrome staining revealed extensive tissue fibrosis, which was most pronounced around the lateral digitiform aneurysm (*Figure 4D*), though fibrosis was also present in several histological sections, including non-irradiated areas (*Figure 4F*).

The IHC was performed to detect apoptosis markers such as p53, Bcl-2, and caspase-3 (*Figure 5B–D*, respectively), with predominantly negative results. The only exception was some areas showing positive staining for caspase-3 in inflammatory cells, a finding considered non-specific.

### Patient 2

A 60-year-old man with CCC and prior oesophagectomy for megaoesophagus (Chagas-related), with an LVEF of 29%, had an ICD for secondary prevention and prior VT ablation. He was admitted with decompensated heart failure and frequent appropriate ICD therapies. Due to recurrent VT during hospitalization, STAR was indicated.

The STAR was performed in April 2022 (25 Gy, 80% isodose, 23 min). The PTV was 82.5 cc, and the ITV was 27.3 cc. The patient improved clinically, with reduced VT burden. However, approximately 9 months after STAR he presented new ICD therapies, and in March 2023, he developed another electrical storm, requiring repeat catheter ablation during the same month. Three VT morphologies were induced—from the right ventricular outflow tract (RVOT; non-irradiated) and basal septal LV (within PTV). The PTV area appeared to have lower bipolar voltage signal. Ablation was applied to both sites.

Initially stable, he was later readmitted with VT recurrence (*Figure 3*) and worsening heart failure, culminating in heart transplantation in March 2024. Notably, the procedure was completed without any surgical complications, such as adhesions or related findings. The anatomical specimen revealed areas of radiofrequency ablation application in the RVOT region (*Figure 2C*), as well as a region of tissue necrosis in the basal septal region of the LV (*Figure 2D* and *E*). This necrosis could potentially be attributed to a steam pop effect resulting from the most recent radiofrequency ablation; however, it was also located within the irradiated area.

**Figure 6:**
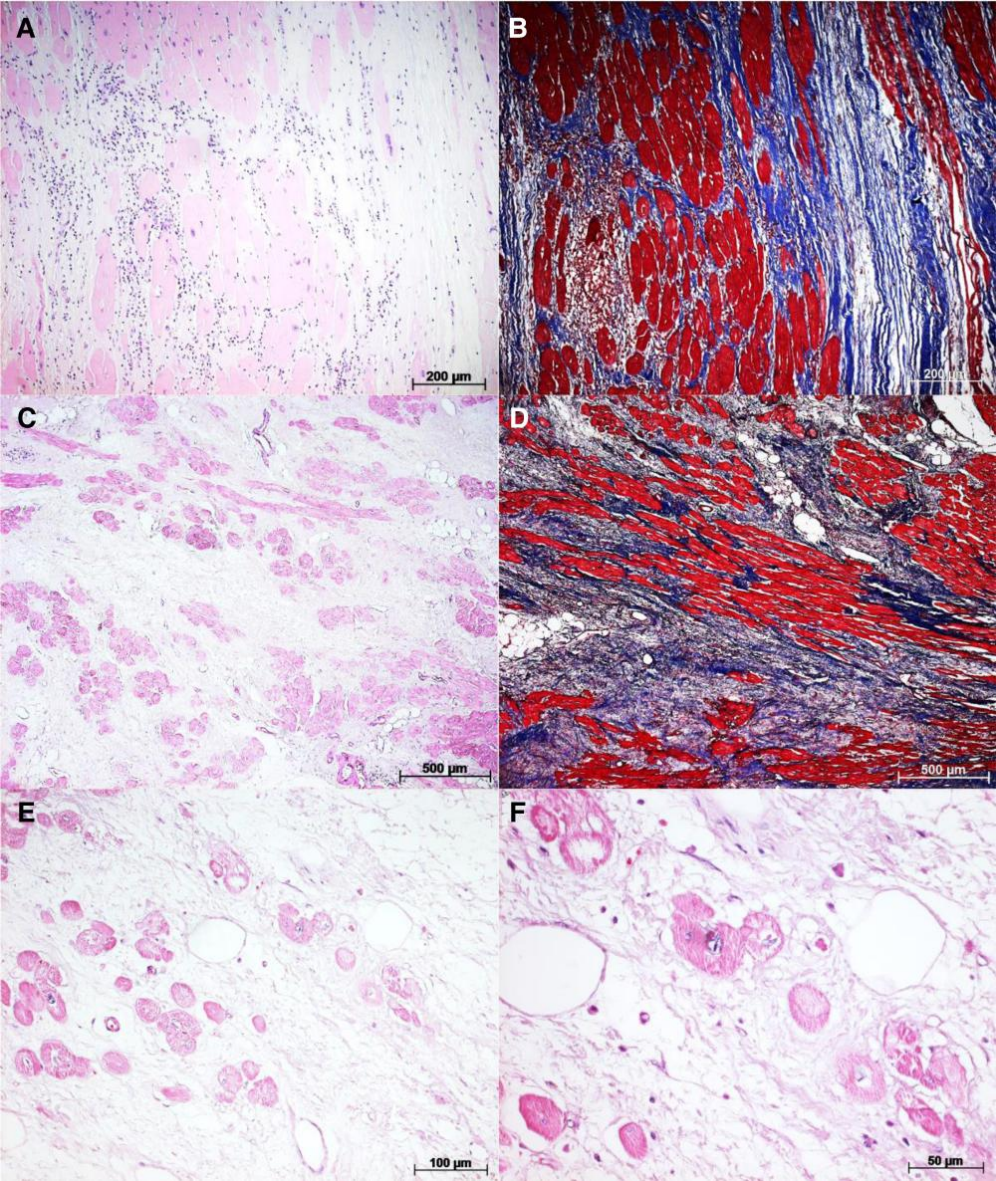
(*A*) Histological section of the non-irradiated area stained with haematoxylin–eosin, showing lymphomononuclear infiltrate associated with fibrotic areas. (*B*) Similar to (*A*), but stained with Masson’s trichrome, highlighting tissue fibrosis (blue). (*C*) Histological section of the irradiated area stained with haematoxylin–eosin; note dense fibrosis with minimal inflammatory infiltrate. (*D*) Masson’s trichrome staining of (*C*), highlighting extensive fibrosis (blue) in the lateral left ventricle wall. (*E*) Irradiated area stained with haematoxylin–eosin at higher magnification, showing myocytolysis and cardiomyocyte vacuolization, with no evident inflammatory infiltrate. (*F*) Similar to (*E*), at even higher magnification.

Histological examination of the non-irradiated (see Supplementary material online, *Figure S2B*) area revealed a lymphomononuclear inflammatory infiltrate interspersed with fibrotic regions (*Figure 6A* and *B*). In contrast, the irradiated area demonstrated dense fibrosis associated with a lower concentration of inflammatory cells (*Figure 6C* and *D*). Structural alterations in cardiomyocytes, such as myocytolysis, were also observed, although they were less frequent compared to the findings in Case 1 (*Figure 6E* and *F*).

**Figure 7:**
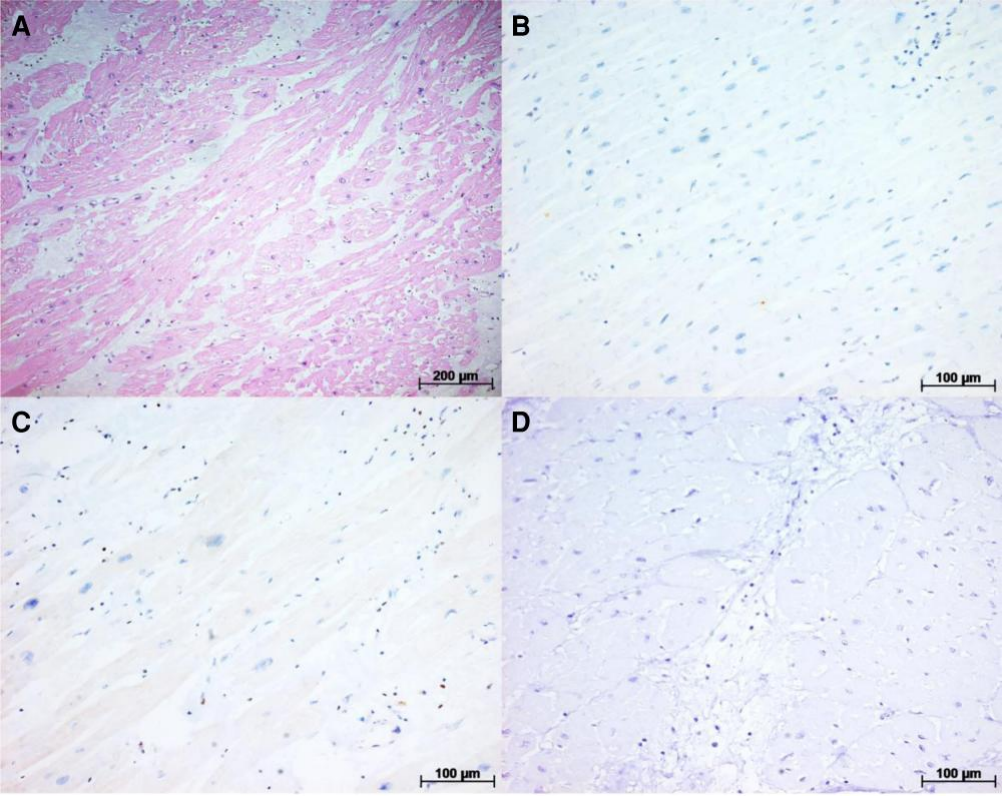
(*A*) Another histological section of the irradiated area stained with haematoxylin–eosin, showing fibrosis associated with mild lymphomononuclear infiltrate. (*B*) Immunohistochemistry for P53—predominantly negative sample. (*C*) Immunohistochemistry for Bcl-2—predominantly negative sample. (*D*) Immunohistochemistry for caspase-3—predominantly negative sample.

The IHC for apoptosis markers p53, Bcl-2, and caspase-3 in the irradiated area yielded negative results (*Figure 7*).

## Discussion

Histopathological data on patients undergoing STAR remain limited and are the focus of ongoing investigation, particularly regarding the timeline of clinical effectiveness. Early case series reported a blanking period of ∼6 weeks, while more recent studies suggest a delayed onset of STAR’s antiarrhythmic effects.^2,3,11^ This delayed response was also seen in our previously reported case of Chagas cardiomyopathy.^4^

Kiani *et al*.^5^ were the first to report STAR-induced changes in four heart transplant recipients, with post-procedure intervals ranging from 12 to 250 days. All cases showed structural cellular damage and alterations in intercellular junctions on electron microscopy, along with varying degrees of fibrosis, generally less extensive than that caused by radiofrequency ablation.

Zhang *et al*.^12^ described early electrical remodelling following STAR, including increased expression of connexin 43 and voltage-gated sodium channels in irradiated myocardial tissue. These changes may enhance conduction velocity and help explain the observed antiarrhythmic effects in the early weeks post-treatment. In fact, STAR case series targeting electrical storm have shown variable efficacy, with response times ranging from 1 to 7 weeks in patients with incessant VT,^13–15^ suggesting that fibrosis is unlikely to be the primary mechanism in the early phase.

Kautzner *et al*.^6^ and Kučera *et al*.^7^ analysed three cases with histological evaluation at 3, 6, and 9 months post- STAR. Caspase-3, a marker of apoptosis, was strongly expressed at 3 months, while dense, well-organized fibrosis with minimal caspase-3 positivity was seen at 9 months. Electrophysiological mapping at 6 months also revealed an expansion of low-voltage areas, supporting a dose-dependent fibrotic response to radiation.^16^

Stereotactic arrhythmia radiotherapy appears to exert part of its therapeutic effect through modulation of the immune response. Emerging evidence indicates that radiation induces a localized inflammatory reaction, activating immune cells that contribute to tissue remodelling and altering the cardiac microenvironment. This response involves the recruitment of lymphocytes, monocytes, and macrophages to irradiated regions,^7,17^ along with conduction disturbances and membrane potential alterations.^18^ These processes may underlie both early tissue injury and later fibrosis.

In both of our cases, we observed extensive myocardial fibrosis with perimysial thickening and focal fibrotic islands, findings characteristic of advanced Chagas cardiomyopathy.^19^ This overlap illustrates the difficulty in distinguishing STAR-induced fibrosis from the baseline fibrotic remodelling seen in this condition, as noted in previous studies.^20^ One limitation is that our fibrosis analysis was only descriptive, not quantitative. Also, we did not perform a histological dose–response evaluation based on the radiotherapy dose fall-off region.

We also identified myocytolysis with cytoplasmic vacuolization of cardiomyocytes—features previously linked to cell death and reported in STAR-treated patients within weeks of therapy.^5,21^ However, immunohistochemical staining for apoptotic markers, including caspase-3, was negative in both cases.

Several factors may explain the absence of caspase-3 positivity. Although tissue samples were selected based on the irradiated area, they were not exhaustive, and focal apoptotic activity may have been missed. Moreover, when compared with the timeline described by Kautzner *et al*.^6^ the biopsy in Patient 1 may have occurred too early to detect apoptotic signalling, while in Patient 2, it may have been too late, after positivity in IHC had subsided.

The recurrence of VT in Patient 2 ∼9 months after STAR, with one of the inducible morphologies originating within the PTV, may indicate a progressive decline in antiarrhythmic efficacy over time. Although previous reports have suggested that STAR may not ensure durable substrate modification in all patients,^22^ no VT recurrences arising from within the PTV have been described in a Swiss cohort.^23^ Whether this represents an isolated case or a phenomenon related to CCC warrants further investigation.

Finally, an important aspect to consider is that we evaluated two cases of STAR in which recurrence occurred, representing a form of negative selection. It is not possible to determine to what extent this may have affected, at least in part, the histological findings typically attributed to STAR.

## Conclusion

The histopathological findings of ventricular myocardium post-STAR in the context of advanced Chagas cardiomyopathy are consistent with those previously described, with findings of acute cellular injury characterized by myocytolysis and cytoplasmic vacuolization within the irradiated area. However, apoptotic markers were negative at the evaluated time frame. As an extensive degree of tissue fibrosis was observed in these advanced Chagas cardiomyopathy patients, with varying degrees of associated inflammatory infiltrate present in both irradiated and non-irradiated areas, this made it challenging to estimate the extent of STAR’s contribution to the fibrotic process. Further studies, including time-course analyses, are needed to better define the immediate and long-term effects of STAR on the arrhythmogenic substrate of Chagas cardiomyopathy.

## Lead author biography


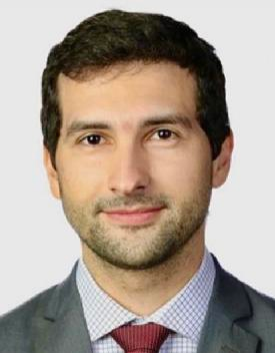


Rodrigo M. Kulchetscki is a cardiologist specializing in clinical arrhythmia, invasive electrophysiology, and cardiac pacing at the Heart Institute (InCor) of the Hospital das Clínicas of the University of Sao Paulo Medical School, where he serves as an attending physician in the Invasive Electrophysiology Laboratory. He is a PhD candidate in cardiology at the same institution and obtained his medical degree from the Federal University of Paraná, Brazil.

## Supplementary Material

ytaf655_Supplementary_Data

## Data Availability

The data underlying this article are largely included within the article and its online Supplementary material. Additional data or materials can be made available upon reasonable request to the corresponding author.
