# Supplementary material for: Pathological findings of stereotactic cardiac radiotherapy for the treatment of ventricular tachycardia in patients with Chagas disease: case series
Source: Eur Heart J Case Rep. 2025 Dec 18;10(1):ytaf655. doi: 10.1093/ehjcr/ytaf655 (PMC12770904; doi:10.1093/ehjcr/ytaf655)

Supplementary Figures EHJ

Supplementary Figure S1

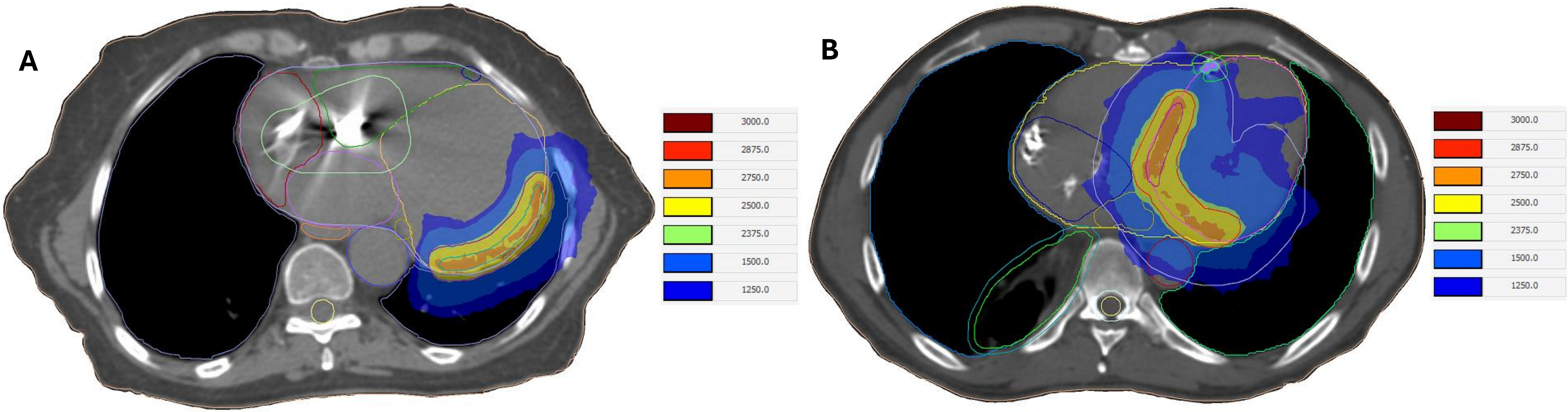

Supplementary Figure S2

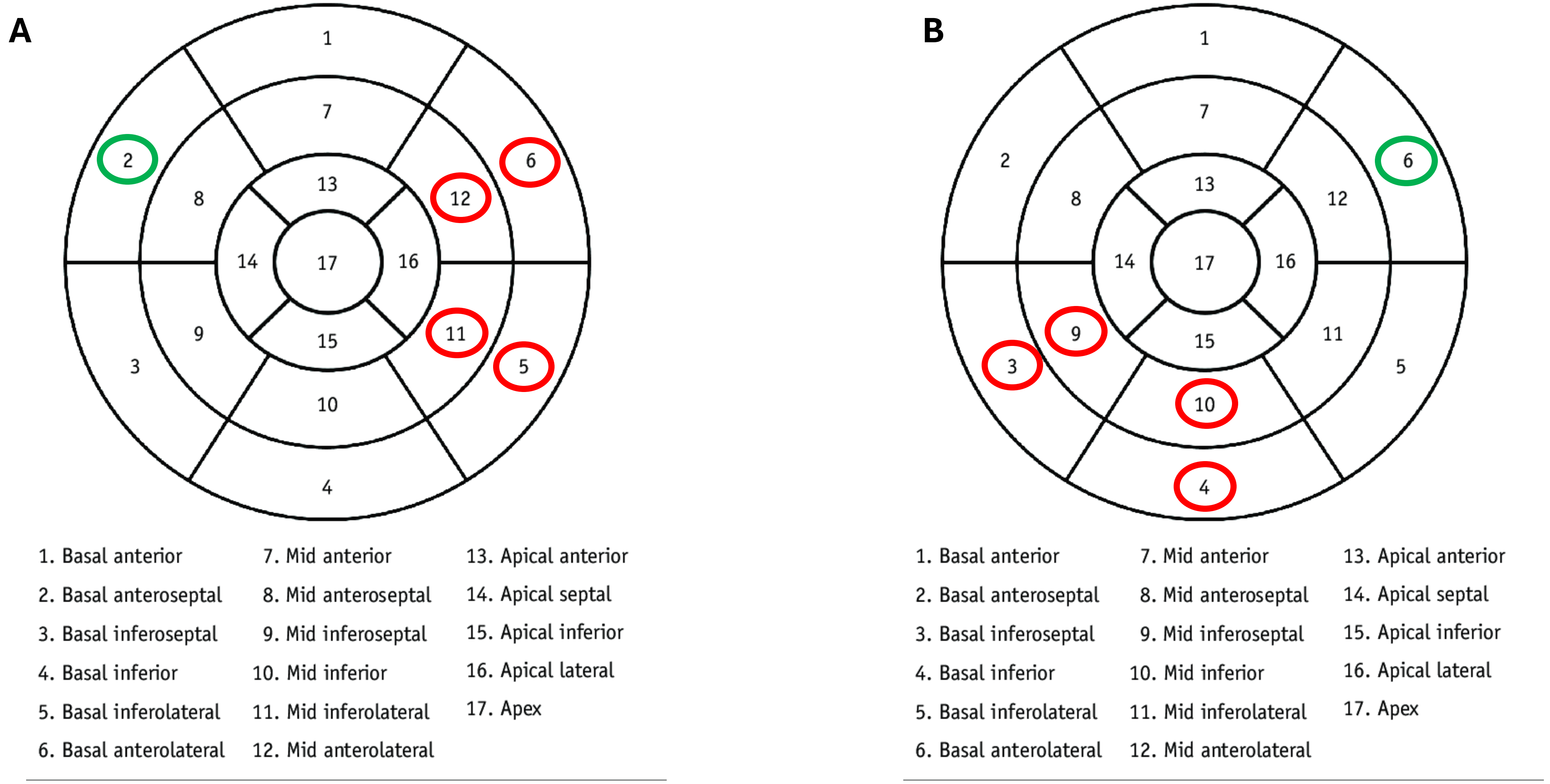

Supplement: ytaf655_Supplementary_Data [file ytaf655_supplementary_data.zip › Figures - Supplement.pdf]
