# Supplementary material for: Pathological findings of stereotactic cardiac radiotherapy for the treatment of ventricular tachycardia in patients with Chagas disease: case series
Source: Eur Heart J Case Rep. 2025 Dec 18;10(1):ytaf655. doi: 10.1093/ehjcr/ytaf655 (PMC12770904; doi:10.1093/ehjcr/ytaf655)
Supplement: ytaf655_Supplementary_Data [file ytaf655_supplementary_data.zip › Supplementary_Legends.pdf]

Supplementary Figure S1

Axial computed tomography images showing the PTV and isodose distribution for STAR in the two patients, (A) Patient 1 and (B) Patient 2. Color-wash represents isodose lines ranging from 1250 cGy (blue) to 3000 cGy (dark red), and color scale is shown to the right of each panel. The 95% isodose line encompasses the PTV in both cases. Note the differences in dose distribution and cardiac anatomical orientation.

Supplementary Figure S2

Segmental distribution of irradiated and non-irradiated myocardial regions in Patients 1 and 2. The diagram illustrates the 17-segment model of the left ventricle. Segments highlighted in red represent regions included in the PTV and exposed to therapeutic radiation. Segments highlighted in green correspond to non-irradiated myocardial areas (defined as receiving <5 Gy) that were selected for histological sampling in each patient. Panel A: Patient 1 – PTV segments: 5, 6, 11, and 12; Non-irradiated segment sampled: 2. Panel B: Patient 2 – PTV segments: 3, 4, 9, and 10; Non-irradiated segment sampled: 6.
